# Driving steady-state visual evoked potentials at arbitrary frequencies using temporal interpolation of stimulus presentation

**DOI:** 10.1186/s12868-015-0234-7

**Published:** 2015-12-21

**Authors:** Søren K. Andersen, Matthias M. Müller

**Affiliations:** School of Psychology, University of Aberdeen, William Guild Building, Aberdeen, AB24 3FX UK; Institute of Psychology, University of Leipzig, Neumarkt 9-19, 04109 Leipzig, Germany

**Keywords:** EEG, SSVEP, Stimulus presentation, Frequency-tagging, BCI, Vision, Oscillation

## Abstract

**Background:**

Steady-state visual evoked potentials have been utilized widely in basic and applied research in recent years. These oscillatory responses of the visual cortex are elicited by flickering stimuli. They have the same fundamental frequency as the driving stimulus and are highly sensitive to manipulations of attention and stimulus properties. While standard computer monitors offer great flexibility in the choice of visual stimuli for driving SSVEPs, the frequencies that can be elicited are limited to integer divisors of the monitor’s refresh rate.

**Results:**

To avoid this technical constraint, we devised an interpolation technique for stimulus presentation, with which SSVEPs can be elicited at arbitrary frequencies. We tested this technique with monitor refresh rates of 85 and 120 Hz. At a refresh rate of 85 Hz, interpolated presentation produced artifacts in the recorded spectrum in the form of additional peaks not located at the stimulated frequency or its harmonics. However, at a refresh rate of 120 Hz, these artifacts did not occur and the spectrum elicited by an interpolated flicker became indistinguishable from the spectrum obtained by non-interpolated presentation of the same frequency.

**Conclusions:**

Our interpolation technique eliminates frequency limitations of the common non-interpolated presentation technique and has many possible applications for future research.

## Background

The steady-state visual evoked potential (SSVEP) is a continuous oscillatory response of the visual cortex which is elicited by a flickering stimulus and has the same temporal frequency as the driving stimulus [1–4]. It can be recorded by electro- or magnetoencephalography and has been widely used in basic and applied research in recent years. In particular, SSVEPs have been employed to study different aspects of selective attention such as spatial attention [5–10], feature-based attention [11–13], visual search [14], competitive stimulus interactions [15–17] and attentional function in healthy old age [18, 19]. Other studies have employed SSVEPs to assess processing of emotional stimuli [20–24] and faces [25, 26], binocular rivalry [27, 28], object processing [29, 30], and figure-ground separation [31]. SSVEPs have also been widely employed in the development of brain-computer interfaces [32, 33].

SSVEPs offer good signal-to-noise ratios, as the signal power is concentrated in a few discrete frequency bands. This allows for easy signal extraction by means of Fourier-transformation if temporally sustained effects are of interest. Dynamic changes in stimulus processing can also be studied by using time–frequency analysis techniques such as Gabor-Filters [34] or wavelets. A particularly powerful application of SSVEPs is the ‘frequency tagging’ technique, in which the cortical processing of multiple stimuli can be assessed simultaneously by flickering each stimulus at a specific frequency [3].

However, as stimulus presentation is necessarily synchronized to the monitor’s refresh rate, the choice of frequencies is severely restricted. For example, when using a monitor with a refresh rate of 60 Hz, a 30 Hz SSVEP can be elicited by presenting the stimulus for one frame and then switching it off for one frame (two frames cycle length) while two frames on and two frames off (four frames cycle length) result in a frequency of 60/4 = 15 Hz. At 60 Hz, this allows for frequencies of 30, 20, 15, 12, 10, 8.57, 7.5 Hz, etc. Using this approach, SSVEPs cannot be elicited at frequencies that are not integer divisors of the refresh rate.

Depending on the purpose of a particular study, the choice of frequencies may be restricted even further. One of the most basic such restrictions is not to use frequencies that are harmonics of other frequencies of concurrently presented stimuli. For example, if one stimulus is presented at 10 Hz, then it is not advisable to use 20 or 30 Hz for a second stimulus, as the harmonic of the 10 Hz stimulus would be superimposed on the SSVEP of the second stimulus. When perceptual differences between stimuli flickering at different rates are to be minimized [9], frequencies must be close to each other, which can only be accomplished at the lower frequencies in our 60 Hz example. Other restrictions apply when a high temporal resolution is required for the question at hand, as for example when studying cued shifts of attention [35–37]. When using Gabor-Filters or wavelets to analyze time-courses at different frequencies, the frequencies should be clearly separated in order to avoid crosstalk. If a Fourier-transform with a short window-length is used, then crosstalk can be avoided by using frequencies that have an integer number of cycles in the chosen time-window (e.g. in a 200 ms window, 10 and 15 Hz have 2 or 3 cycles, respectively). In other cases, it may be important to separate SSVEPs from frequency bands of the EEG carrying other signals, such as transient ERPs or the alpha-band, by choosing appropriate frequencies for the SSVEP. Last but not least, in some cases it might be desirable to use stimuli with equal on–off rations in order to equate stimulus luminance over time. If, for example, all stimuli are to have a 50/50 on–off ratio, then frequencies are limited to even integer divisors of the refresh rate.

Whatever the particular reasons for the choice of frequencies in an SSVEP study, any additional criteria further reduce the already limited number of potential frequencies. Especially in frequency-tagging studies with multiple stimuli presented at different frequencies, the practically available set of frequencies may be far from the theoretically ideal number and choice of frequencies. This is particularly the case in the development of brain-computer interfaces (BCIs), which allow a person to convey his or her intentions by attending to a particular stimulus. Here, the information transfer rate can be improved with higher numbers of stimuli (i.e. frequencies) and certain applications, such as spelling or typing numbers, ideally require a large number of stimuli [38].

To surpass these limitations, an approximation technique for stimulus presentation has recently been proposed, which allows for driving SSVEPs at frequencies that are not limited to integer divisors of the monitor’s refresh rate [39]. This technique interleaves stimulus sequences of different length to approximate a specific presentation rate. For example, a rate of 11 Hz would be approximated at a refresh rate of 60 Hz by interleaving cycles of five or six frames length (corresponding to 12 and 10 Hz, respectively). This approximation approach is successful in eliciting SSVEPs at the desired frequencies which are largely comparable to those that would be elicited by a traditional stimulation technique. However, it also elicits additional signal power at other interference frequencies [38]. Such artifacts of the stimulation technique may be irrelevant for some approaches, in which the entire analysis is limited to a few specific frequencies at which no interference occurs. However, for other applications such interference artifacts may be unacceptable. If, for example, the goal of a study is to concurrently assess SSVEPs and activity in naturally occurring frequency bands of the EEG (e.g. theta-, alpha- or gamma-band), then this frequency band should not be contaminated by such interference artifacts.

We here devised and tested an interpolation technique in order to drive SSVEPs at arbitrary frequencies that can be chosen independently of the screen refresh rate. Ideally, a stimulus interpolation technique should (1) robustly produce signal power at the desired frequency (and potentially its harmonics) and (2) not produce additional signal power in other frequency bands. In order to test our technique against these criteria, we presented stimuli flickering at four different frequencies using two different monitor refresh rates. This was done in such a way, that each frequency was elicited in the standard non-interpolated way at one refresh rate, while being interpolated at the other refresh rate.

If our technique satisfies the two above criteria, then the spectrum elicited when a frequency is driven non-interpolated at one refresh rate should be indistinguishable from the spectrum elicited when the same frequency is driven by interpolated stimulation at another refresh rate.

## Methods

### Subjects

Fifteen right-handed subjects (10 female, ages 19–30, average 23.8 years) with normal or corrected-to-normal visual acuity participated in the experiment after giving informed consent and received either a small financial bonus (6 € per hour) or credit points. All subjects were included in the statistical analysis. The study was conducted in accordance with the Declaration of Helsinki and the guidelines and requirements for electrophysiological studies of the ethics committee of the University of Leipzig.

### Interpolation technique

**Fig. 1:**
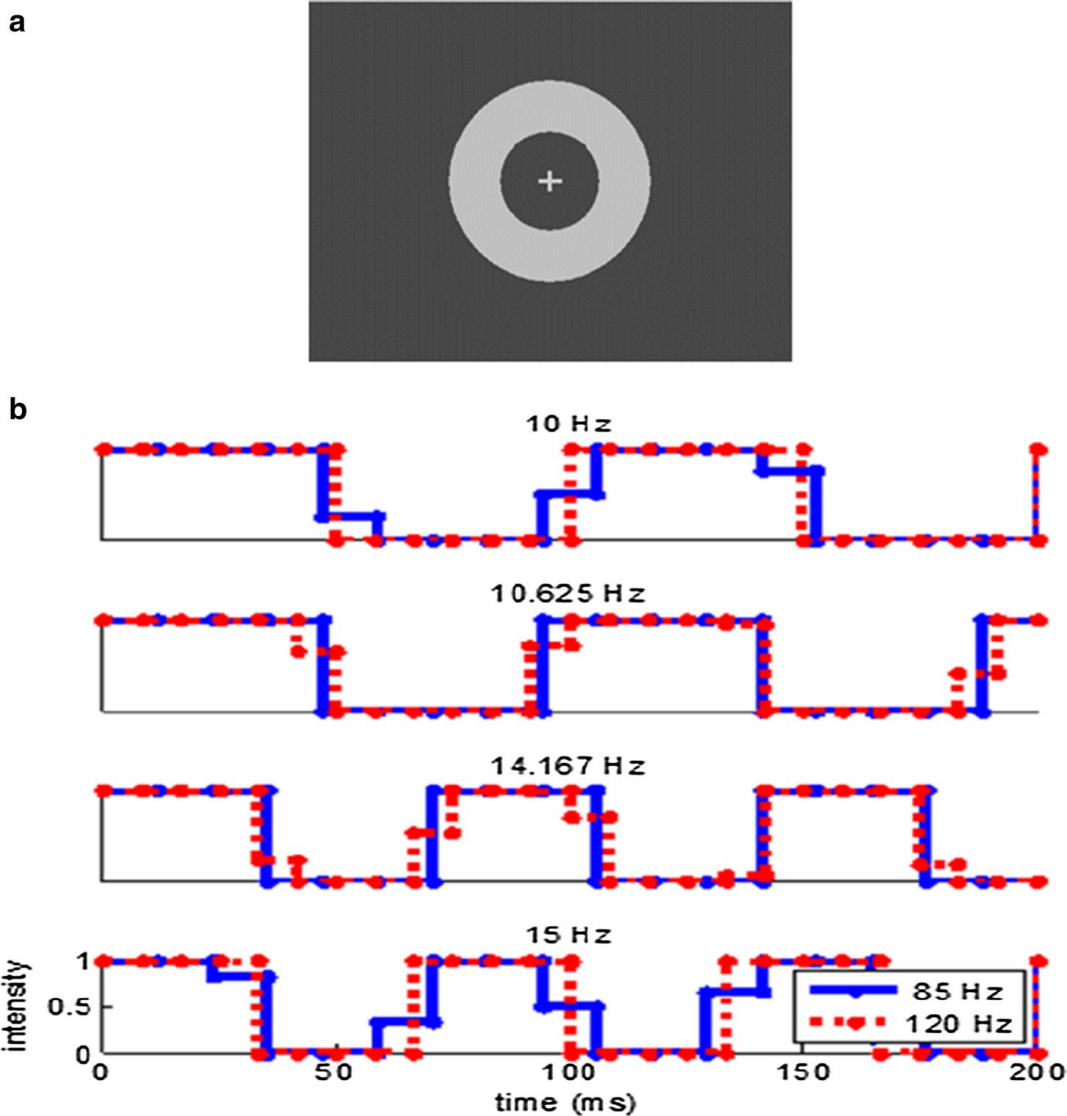
**a** Stimulus display. Participants performed a simple detection task at fixation while SSVEPs elicited by the flickering ring were recorded **b** Illustration of stimulus intensity as a function of time for the four employed frequencies at each of two monitor refresh rates. 10.0 and 15.0 Hz waveforms were non-interpolated at a refresh rate of 120 Hz and interpolated at 85 Hz. 10.625 and 14.167 Hz were non-interpolated at 85 Hz and interpolated at 120 Hz

A specific SSVEP-frequency can be elicited by presenting a stimulus for a certain number of frames and then switching it off for another number of frames. The elicited frequency is then equal to the monitor refresh rate divided by the total cycle length (number of frames ‘on’ plus number of frames ‘off’). For example, at a refresh rate of 120 Hz, a 10.0 Hz SSVEP can be elicited by flickering a stimulus with a cycle length of 12 frames (e.g. 6 frames on, 6 frames off). However, if the monitor refresh rate is 85 Hz, it is not possible to elicit an SSVEP at 10.0 Hz using this approach, as this would require a cycle length of 85/ 10 HZ = 8.5 frames. Using a 50/50 on–off ratio, this would require the stimulus to be on and off for 4.25 frames respectively, which is technically not possible. However, this can be approximated by presenting the stimulus at full intensity for 4 frames and then presenting it at 25 % intensity for one frame (4 + 0.25 = 4.25 frames on). After this, the stimulus would be off for another 3 frames and then be presented at 50 % intensity (0.75 + 3+0.5 = 4.25 frames off). So the impossible requirement to present a stimulus for a fractional duration of frames at each on–off reversal is approximated by presenting the stimulus for a whole frame at an intermediate intensity. Using this interpolated stimulation technique the time-averaged intensity of the stimulus remains unchanged. However, the technically impossible sharp transitions from on to off at time-points that do not coincide with the monitor’s refresh rate are replaced by transitions synchronous to the monitor’s refresh rate at intermediate stimulus intensities (Fig. 1b).

In the general case, our interpolation technique is defined as follows. With a monitor refresh rate R and a desired stimulus frequency f, the required cycle length λ is defined by:

1 $$\lambda = R/f$$

The stimulus intensity w (1:on, 0:off) in any given frame i can then be calculated as follows:

2 $$w = \left\{ {\begin{array}{*{20}l} {1,} \hfill & {if\;1 \le i\bmod \lambda \le r_{on} \lambda } \hfill \\ {r_{on} \lambda + 1 - i\bmod \lambda ,} \hfill & {if\;r_{on} \lambda < i\bmod \lambda < r_{on} \lambda + 1} \hfill \\ {0,} \hfill & {if\;r_{on} \lambda \le i\bmod \lambda } \hfill \\ {i\bmod \lambda ,} \hfill & {if\;i\bmod \lambda < 1} \hfill \\ \end{array} } \right.$$

where r_on_ denotes the fraction of the stimulus cycle in which the stimulus is on (r_on_ = 0.5 in the recordings reported here). Note that i modulo λ is the position within the current flicker cycle. The first line defines the frames in which the stimulus is on at full intensity, the second line the on–off transitions, the third line the off-frames and finally the fourth line defines the off–on transitions.

In order to present stimuli at intermediate intensities 0 < w <1 two further things need to be taken into account. First, the off-phase of a stimulus is not necessarily black but could be any arbitrary color C_off_ and thus intermediate stimulus intensities C must be a weighted average of the stimulus color C_on_ and the background or ‘off’ color C_off_. Second, the relationship between color values in a stimulation program V_in_ (e.g. RGB values) and the output of a computer monitor V_out_ is not linear, but defined by a power function with an exponent gamma (γ):

3 $$V_{out} \propto V_{in}^{\gamma }$$

The exponent γ depends on the specific graphics hardware and its settings and usually lies between 1.8 and 2.2 for standard computer equipment. Taking the two points above into account, the output color C can be computed as:

4 $$C = \left( {wC_{on}^{\gamma } + \left( {1 - w} \right)C_{off}^{\gamma } } \right)\!{^{1}\!/\!{\gamma}}$$

To test this interpolation technique, we recorded the EEG elicited by a ring flickering at four different frequencies in different conditions. The four frequencies were chosen within a frequency range commonly employed in SSVEP experiments in such a way that two of them could be presented non-interpolated at a monitor refresh rate of 85 Hz, but not at 120 Hz (10.625 and 14.167 Hz), while the two other frequencies could be presented non-interpolated at a monitor refresh rate of 120 Hz, but not at 85 Hz (10 and 15 Hz, see Fig. 1). If the differences introduced by the interpolation technique are sufficiently subtle to be imperceptible to the human brain, then the EEG elicited by interpolated and non-interpolated stimulation of the same frequency should be indistinguishable. Flicker at 60 Hz elicits robust phase locked activity in the primary visual cortex of humans and macaque monkeys [40], however the magnitude of such activity in humans is already strongly reduced at 72 Hz [41]. In light of these findings, we considered it unlikely that distortions produced by stimulus interpolation would be imperceptible at monitor refresh rates of 72 Hz or less. Therefore, we chose a somewhat higher frequency of 85 Hz as the lower refresh rate for our experiment and a rate of 120 Hz as the higher refresh rate, since this was the maximum rate available with our equipment.

### Stimulus material and procedure

Stimulation was presented on a 19″ Belinea 10 60 75 cathode ray tube (CRT) monitor set to a resolution of 640 × 480 pixels and 32 bits per pixel color mode. At a viewing distance of 80 cm, the ring had an outer diameter corresponding to 8.1° degrees of visual angle and an inner diameter of 4.06°. The bars of the fixation cross had a length corresponding to 0.81° × 0.16°. The background had a luminance of 9.4 cd/m^2^ and the fixation cross and flickering ring had a luminance of 79.7 cd/m^2^ (Fig. 1a).

To ensure correct intermediate luminance values for interpolated flicker, luminance calibration and gamma correction was performed separately for 85 and 120 Hz refresh rates. This is important, as the same RGB color values do not generally produce the same luminance values for different screen modes. We empirically estimated γ by presenting different gray values statically and measuring their luminance, resulting in γ = 2.0 for both refresh rates. Stimulation was realized using Cogent Graphics (John Romaya, LON at the Wellcome Department of Imaging Neuroscience). In each trial, the flickering ring was presented for approximately 3340 ms (284 frames at 85 Hz and 401 frames at 120 Hz). Timestamps for each frame of stimulus presentation were stored in order to verify the accuracy of stimulus timing. We did not detect any dropped frames throughout the entire dataset.

To control the allocation of attention, participants performed a simple target detection task at fixation. Beginning from 300 ms after stimulation onset (after 26 frames at 85 Hz or 36 frames at 120 Hz), the length of either the horizontal or vertical bar of the fixation cross could briefly change by 0.12° of visual angle. Length decrements of either bar were defined as targets and required a detection response by pressing space bar. Responses to analogous length increments constituted false alarms. The duration of targets and distractors was about 50 ms (4 frames at 85 Hz or 6 frames at 120 HZ). Any combination of up to three targets and/or distractors could occur within a single trial and the onsets of successive targets or distractors were separated by at least 700 ms (59 frames at 85 Hz or 84 frames at 120 Hz). Responses occurring from 200 ms to 850 ms after the onset of targets or distractors were counted as hits or false alarms, respectively. Over the entire experiment, a total of 45 targets and 45 distractors were presented for each of the eight conditions (4 frequencies each presented at two different monitor refresh rates).

The experiment consisted of 480 trials (60 per condition) presented in 8 blocks of 60 trials each. Trials of all conditions were presented in random order with the constraint that any single block contained only trials presented at the same monitor refresh rate. This was done to prevent frequent switching of the screen mode. Blocks at 85 and 120 Hz monitor refresh rate were presented in random order. Responding hand was switched after the first 4 Blocks. Prior to recordings, participants performed one or two blocks of task training (average 1.3 blocks).

### EEG recordings and analysis

Brain electrical activity was recorded non-invasively at a sampling rate of 256 Hz from 64 Ag/AgCl electrodes mounted in an elastic cap using an ActiveTwo amplifier system (BioSemi, Amsterdam, The Netherlands). Lateral eye movements were monitored with a bipolar outer canthus montage (horizontal electroocculogram). Vertical eye movements and blinks were monitored with a bipolar montage positioned below and above the right eye. Processing of EEG data was performed using the EEGLab toolbox [42] in combination with custom written procedures in Matlab (The Mathworks, Natick, MA, USA).

Analysis epochs were extracted from 100 ms before to 3400 ms after stimulation onset. All epochs were detrended (removal of mean and linear trends) and epochs with eye movements or blinks were rejected from further analysis. All remaining artifacts were corrected or rejected by means of an automated procedure using a combination of trial exclusion and channel approximation based on statistical parameters of the data [43]. This led to an average rejection rate of 10.6 % of all epochs, which did not differ between conditions. Subsequently, all epochs were re-referenced to average reference and averaged for each experimental condition.

**Fig. 2:**
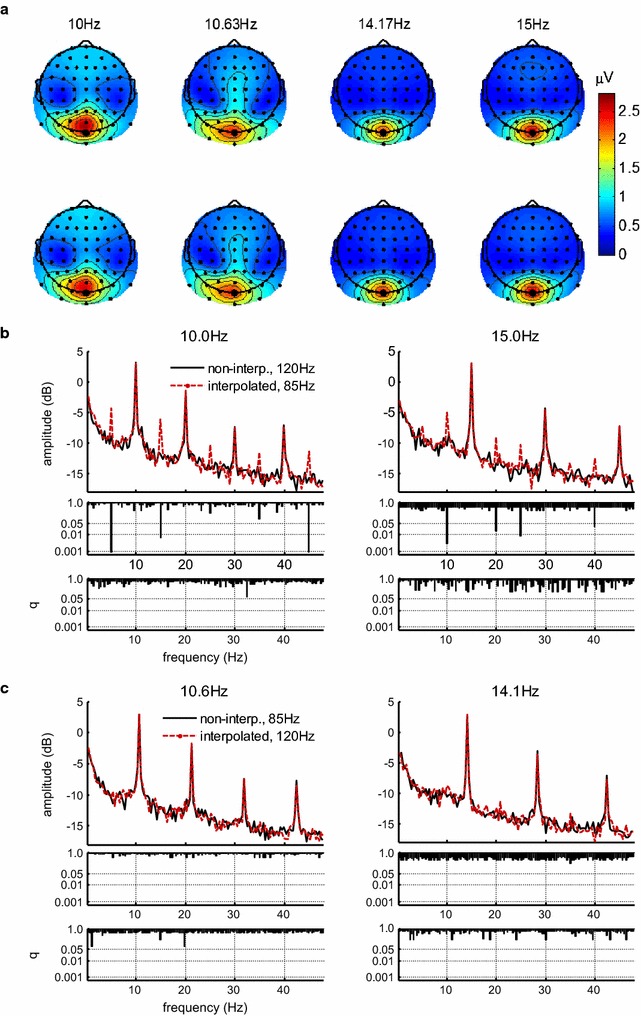
**a** Isocontour voltage maps of SSVEP amplitudes averaged over all subjects at the four stimulation frequencies for non-interpolated (*top-row*) and interpolated (*bottom-row*) stimulation; **b** grand-average spectrum (*top*) and q-values comparing amplitudes (*middle*) elicited by interpolated vs. non-interpolated stimulation at a refresh rate of 85 Hz reveal clear artifacts of the interpolation technique. A comparison of the variance of the spectrum elicited by interpolated vs. non-interpolated stimulation (*bottom*) reveals no differences, indicating that artifacts were elicited consistently across participants; **c** no artifacts were apparent when stimulation was interpolated at a refresh rate of 120 Hz. 0 dB corresponds to 1 µV

To quantify the spectral content in each condition, analysis windows were defined that contained an integer number of cycles of the driven SSVEP frequency (28, 30, 40 and 42 cycles for 10.0, 10.6 14.1 and 15.0 Hz, respectively). All analysis windows began 400 ms after stimulation onset in order to exclude the evoked potential to stimulation onset and to allow the SSVEP sufficient time to build up. The resulting analysis windows lasted from 400 to 3200 ms (10.0 and 15.0 Hz) or to 3225 ms (10.6 and 14.1 Hz) after stimulation onset. The amplitude spectrum of the data in these time windows was obtained by taking the absolute value of the complex Fourier coefficients for each frequency and electrode separately. Iso-contour voltage maps of amplitudes at the frequencies of the elicited SSVEPs for each condition showed a narrow peak over occipital electrodes (Fig. 2a). Accordingly, electrode Oz was chosen for statistical analysis.

In the following step, we compared the recorded amplitude spectrum between 0 and 48 Hz for each of the four pairs of conditions with the same SSVEP frequency (e.g. 10 Hz non-interpolated was compared to 10 Hz interpolated). In order to better encompass the large amplitude differences in this wide frequency band, all amplitudes were transformed to a decibel scale by taking the logarithm to a base of 10 and multiplying by 10 (e.g. 1 µV corresponds to 0 dB, 0.1 µV correspond to −10 dB, see Fig. 2b). The amplitudes (in dB) for each frequency above 0 Hz and below 48 Hz were compared by means of paired t-tests. Due to slightly different window lengths, this analysis comprised 134 different frequencies for 10.0 and 15.0 Hz and 135 frequencies for 10.6 and 14.1 Hz. To account for multiple comparisons, we controlled the false discovery rate (FDR) [44] by correcting p-values using the ‘mafdr’ function in Matlab (The Mathworks). The resulting q-values (i.e. corrected p-values) depicted in Fig. 2b are significant when they are smaller than or equal to 0.05. In order to test for possible differences in the variance between participants of the recorded spectrum, a Bartlett test was conducted analogously to the t-tests described above and using the same correction for multiple comparisons.

## Results

### Behavioral data

The behavioral performance of participants is displayed in Table 1. Participants performed the task reliably with average hit-rates above 90 %, average false alarm rates around 10 % and average reaction times just below 500 ms. One-factorial repeated measures ANOVAs using Greenhouse-Geyser Correction for non-sphericity were conducted separately for hit rates, false alarm rates and reaction times. None of the three measures of behavioral performance differed between experimental conditions [hit rate: F(3.691, 51.571) = 1.325; false alarm rate: F(2.880, 40.320) = 0.361; reaction time: F(4.019, 56.263) = 2.022; all p > 0.1].

**Table 1:** Mean hit and false alarm rates and reaction times for all conditions

Condition	Hits	False alarms	Reaction time
10.0 Hz at 85 Hz (Interp.)	93.2 (1.8) %	10.5 (3.4) %	495.3 (7.9) ms
10.6 Hz at 85 Hz (Non-interp.)	93.3 (1.1) %	10.4 (2.7) %	493.7 (8.9) ms
14.1 Hz at 85 Hz (Non-interp.)	92.0 (1.7) %	10.8 (3.4) %	494.5 (6.2) ms
15.0 Hz at 85 Hz (Interp.)	92.0 (1.8) %	10.7 (3.3) %	506.2 (9.9) ms
10.0 Hz at 120 Hz (Non-interp.)	93.3 (1.4) %	10.2 (2.6) %	490.5 (8.7) ms
10.6 Hz at 120 Hz (Interp.)	95.0 (1.2) %	8.9 (2.2) %	489.9 (8.1) ms
14.1 Hz at 120 Hz (Interp.)	92.7 (1.4) %	9.9 (2.9) %	486.2 (7.1) ms
15.0 Hz at 120 Hz (Non-interp.)	94.4 (1.2) %	11.1 (3.0) %	490.4 (7.0) ms
Average	93.2 (1.2) %	10.3 (2.8) %	493.3 (7.0) ms
F	1.325	0.361	2.022
p	0.274	0.774	0.103
η^2^	8.6 %	2.5 %	12.6 %

Values in brackets indicate standard errors of the mean. None of the three measures of behavioral performance differed between experimental conditions

### Electrophysiological data

Amplitude spectra and statistical results are depicted in Fig. 2. For each of the four SSVEP frequencies, the spectrum elicited with non-interpolated stimulation was compared against the spectrum elicited with interpolated stimulation.

For 10.0 Hz, interpolated stimulation presented at a monitor refresh rate 85 Hz elicited clear additional peaks in the spectrum, that were not present when 10 Hz SSVEPs were elicited by non-interpolated flicker at a refresh rate of 120 Hz (Fig. 2b). Pairwise t-tests for each frequency in the spectrum and corrected for multiple comparisons using the false discovery rate revealed significantly higher amplitudes at 5.0 Hz [t(14) = −6.550, q = 0.000845], 15.0 Hz [t(14) = −5.089, q = 0.00632] and 44.9 Hz [t(14) = −6.471, q = 0.000845]. Although additional peaks are also visible at 25 and 35 Hz, these were not significantly different from the amplitudes elicited with non-interpolated stimulation (see Fig. 2b).

Additional peaks were also visible in the spectrum when SSVEPs were elicited at 15.0 Hz using interpolated flicker at a refresh rate of 85 Hz, as compared to when 15.0 Hz SSVEPs were elicited by non-interpolated flicker at a refresh rate of 120 Hz. Significantly higher amplitudes were observed at 10.0 Hz [t(14) = −5.857, q = 0.00317], 20.0 Hz [t(14) = −4.263, q = 0.0200], 25.0 Hz [t(14) = −4.840, q = 0.00997] and 39.9 Hz [t(14) = −3.815, q = 0.0360)].

The variance across participants did not differ significantly between interpolated and non-interpolated stimulation at any point of the spectrum for any of the four SSVEP frequencies (Fig. 2b, c, bottom panels). This indicates that the pattern of presence or absence of interpolation artifacts was consistent across participants, because the presence of such artifacts in only some participants would have led to a higher variance than in the non-interpolated control condition.

**Fig. 3:**
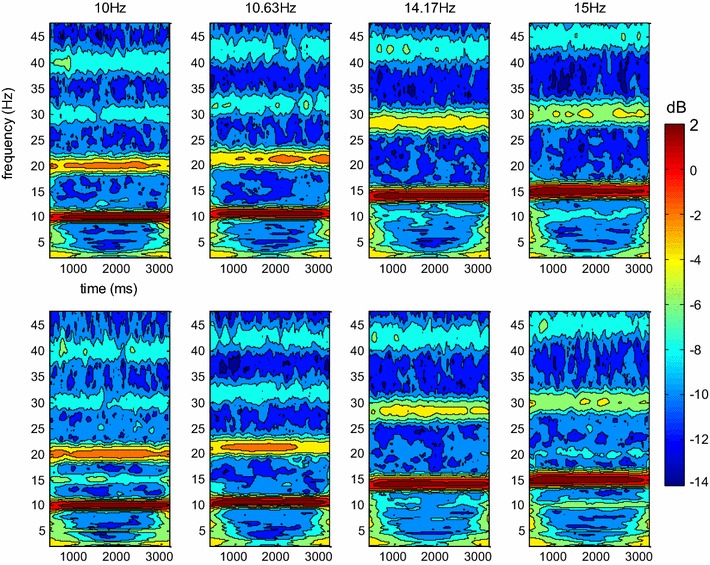
Spectrograms of the recorded data averaged over all subjects at the four stimulation frequencies for non-interpolated (*top-row*) and interpolated (*bottom-row*) stimulation. Additional signal power was elicited in specific frequency bands when stimulus presentation was interpolated at a refresh rate of 85 Hz (10 and 15 Hz), but not at a refresh rate of 120 Hz (10.63 and 14.17 Hz). 0 dB corresponds to 1 µV

To provide a more detailed depiction of the recorded data, the grand mean spectrograms were computed for each condition by means of a bank of Gabor-Filters [34] located at the 134 (10.0 and 15.0 Hz) or 135 (10.6 and 14.1 Hz) frequencies of the spectrum used for the main analysis (Fig. 3).

In summary, interpolated stimulation at a refresh rate of 85 Hz successfully elicited robust SSVEPs at the stimulated frequencies, which did not differ from those elicited by non-interpolated flicker. However, additional signal power was also elicited at other frequencies, which represents an artifact of the interpolation technique. A different picture emerged when SSVEPs were elicited at 10.6 and 14.1 Hz by interpolated stimulation with a refresh rate of 120 Hz. Here, the spectrum was indistinguishable from the one elicited by non-interpolated stimulation of the same frequencies at 85 Hz (Fig. 2c). The paired comparison yielded no significantly different amplitudes for any of the tested frequencies.

## Discussion

We investigated the feasibility of eliciting SSVEPs by using an interpolated stimulation technique in order to overcome the restrictions of available frequencies inherent to no-interpolated stimulation. To verify the adequacy of our method, we compared the recorded spectrum to a control condition in which the same frequency was elicited at another monitor refresh rate without interpolation. An ideal interpolation technique should elicit spectra that are indistinguishable from those recorded without interpolation. Our results consistently showed that this was not the case for interpolation using a monitor refresh rate of 85 Hz: although the elicited SSVEP response in these conditions did not differ from recordings without interpolation, additional peaks at other frequencies were elicited which constitute an unwanted artifact of the interpolation. This was however not the case for interpolation at a refresh rate of 120 Hz, where amplitudes at the elicited SSVEP frequencies and all other frequencies were comparable to those elicited by non-interpolated stimulation. Thus it seems that the temporal resolution of processing in the early visual areas that have previously been identified as the sources of the SSVEP [12, 16, 45] is still high enough to detect the differences in interpolated flicker when presented at a refresh rate of 85 Hz, but not at 120 Hz.

We used our interpolation technique to approximate a square wave luminance flicker at high contrast. The elicited spectrum was indistinguishable from the one elicited by non-interpolated flicker when a refresh rate of 120 Hz, but not 85 Hz, was employed. This implies that a refresh rate of 120 Hz is sufficient to interpolate stimulus presentation with our technique without eliciting unwanted artifacts in the spectrum. It should be noted that we tested our technique under ‘unfavorable’ conditions that were likely to reveal artifacts in the spectrum. Such artifacts may be less evident at lower stimulus contrast or if a smoother stimulus waveform than a square wave (e.g. a sine wave) was approximated. Also, luminance flicker can be perceived at higher frequencies than chromatic flicker [46]. Therefore, interpolating the flicker of chromatic stimuli of equal luminance as the background should produce less artifacts, and may thus be feasible at refresh rates lower than 120 Hz.

Other authors have recently proposed a different approximation technique to elicit SSVEPs at frequencies independent of the monitor refresh rate [38, 39]. This technique elicits robust SSVEPs at the desired frequencies and is slightly simpler to implement than ours, as it does not require intermediate stimulus intensities. However, our technique is superior in that it does not produce artifacts at interference frequencies as long as a refresh rate of 120 Hz is utilized. This difference may be critical for applications that also measure activity in other frequency bands (e.g. alpha, gamma) than those at which SSVEPs are elicited or when ERPs to discrete events interleaved in the SSVEP stimulation are to be measured.

We tested our interpolation technique for frequencies in the range between 10 and 15 Hz, which is often utilized in SSVEP experiments. SSVEPs can be elicited at lower frequencies too. However the frequency range below 10 Hz is of less interest for interpolated stimulation, because non-interpolated stimulation already offers much more flexibility in this range. For example, at a refresh rate of 120 Hz, the narrow frequency band between 4 and 6 Hz allows for 11 different frequencies to be driven using non-interpolated stimulation. Our interpolation technique is of high utility in cases where temporal changes in the processing of multiple stimuli are of interest. In such cases, higher frequencies are preferable because they allow for better temporal resolution. However, even at a refresh rate of 120 Hz, the wide frequency band from 10 to 60 Hz only contains 11 different frequencies that can be elicited by non-interpolated stimulation. Of these, five frequencies are higher harmonics of other frequencies, thus only leaving 6 frequencies that can be utilized together. Once other constraints are taken into consideration (e.g. equal on–off ratios, sufficient separation in frequency-space to avoid crosstalk, avoiding specific frequency bands; see introduction), the availability of frequencies is reduced even more thus rendering experiments that aim to investigate rapid dynamic changes in the processing of more than 2–3 stimuli infeasible without temporal interpolation of stimulus presentation.

It should be noted that our interpolation technique is in not limited to the simple black and white stimuli employed here. By applying Eq. (4) to the red, green and blue (RGB) values of a stimulus separately, this procedure can be utilized for flickering stimuli of any arbitrary color against a background of any arbitrary color. Furthermore, stimuli do not have to be of uniform color. For example, a bitmap image flickering against a background (or two alternating bitmap images) can be implemented by applying Eq. (4) to each pixel separately. Also, the phase of the stimulation can be shifted by any desired angle φ (0 < = φ < 2π). If we define j as

5 $$j = \left( i + \lambda \phi/ 2\pi \right)\bmod \lambda$$

and insert j into Eq. (2). Stimulus intensity w of a waveform shifted by an arbitrary phase φ is given by

6 $$w = \left\{ {\begin{array}{*{20}l} {1,} \hfill & {if\;1 \le j \le r_{on} \lambda } \hfill \\ {r_{on} \lambda + 1 - j,} \hfill & {if\;r_{on} \lambda < j < r_{on} \lambda + 1} \hfill \\ {0,} \hfill & {if\;r_{on} \lambda \le j} \hfill \\ {j,} \hfill & {if\;j < 1} \hfill \\ \end{array} } \right.$$

In conclusion, we devised and tested an interpolation technique that, using Eq. (6) allows to drive SSVEPs at any frequency up to half the monitor’s refresh rate with arbitrary on/off ratio (as defined by r_on_) and phase φ. At refresh rates of 120 Hz, this interpolation technique elicited SSVEPs that are indistinguishable from those elicited by non-interpolated flicker without producing any detectable artifacts in other parts of the spectrum. This technique thus portends many future applications in both basic and applied research.
